# Influenza Myocarditis: A Literature Review

**DOI:** 10.7759/cureus.12007

**Published:** 2020-12-10

**Authors:** Nischit Baral, Prakash Adhikari, Govinda Adhikari, Sandip Karki

**Affiliations:** 1 Internal Medicine, McLaren Flint/Michigan State University College of Human Medicine, Flint, USA; 2 Internal Medicine, Piedmont Athens Regional Medical Center, Athens, USA; 3 Internal Medicine, McLaren Flint/Michigan State University, Flint, USA

**Keywords:** influenza virus type a and b, myocarditis, viral myocarditis, myopericarditis

## Abstract

Viral myocarditis is not uncommon but the role of the influenza virus in causing myocarditis is less studied. It is difficult to diagnose influenza myocarditis. Due to bacterial and viral co-infection during influenza outbreaks, it becomes more difficult to distinguish influenza myocarditis from other causes. Our article provides current information on influenza myocarditis. We did a literature search using appropriate terms and reviewed articles published by November 2020. Our study highlights the incidence of influenza myocarditis and the need to become aware of this condition, especially during epidemics and pandemics. Our study highlights that although influenza myocarditis is a rare condition, it can be fatal. There should be increased awareness about the condition. By the early diagnosis and treatment of influenza myocarditis, we can prevent fatal complications.

## Introduction and background

Influenza viruses are members of ribonucleic acid (RNA) viruses belonging to the orthomyxoviridae family. There are mainly four members of influenza viruses - A, B, C, and D - based on antigenic differences. They can be divided into subtypes based on 16 hemagglutinin (HA) (H1-H16) and nine neuraminidase (NA) (N1-N9) surface proteins. HA protein helps in the attachment to host cellular receptors during the process of infection. NA is involved in the release of viral progeny from the cell surface. These viruses have a segmented genome. This gives them the ability to undergo mutation and recombination to produce new strains. The origin of new strains offers new characteristics and pathogenicity and often coincides with seasonal epidemics and global pandemics [1].

Influenza past, present, and future

Influenza virus is the cause of major pandemics in the past: the Spanish flu in 1918 caused by H1N1, Asian flu in 1957 by H2N2, Hong Kong flu in 1968, and swine flu in 2009-2010 by H1N1 [2-6]. Seasonal epidemics are common with influenza A and B viruses and never with influenza C and D. Influenza type C infection is generally limited to mild upper respiratory illness below age six [7]. Influenza D virus primarily affects cattle and does not affect humans [8]. Data from the World Health Organization and partners found that globally there were more than 200,000 respiratory deaths (range 105,700-395,600) and more than 100,000 cardiovascular deaths (range 46,000-179,900) associated with the 2009 pandemic influenza A H1N1 [6]. During the 2017-2018 flu season, US Centers for Disease Control and Prevention (CDC) estimated 959,000 hospitalizations and 79,400 deaths from influenza. The same year, 48.8 million people got an infection with Influenza and 22.7 million people seek healthcare. This estimated disease burden is the highest after the 2009 pandemic, even after the introduction of highly effective annual influenza vaccine. Cardiovascular complications are the second most common cause of death due to influenza [9]. Other reported complication involves a combination of the cardiovascular and respiratory system (influenza pneumonia and secondary bacterial pneumonia), central nervous system (meningitis, encephalitis, encephalopathy) and musculoskeletal system (myositis) [6,10-11]. CDC ranks influenza number eighth on the leading top 10 causes of death in the USA. Furthermore, influenza-related complications are common in extremes of age (children <5, adults> 65), pregnant female, immunocompromised states like human immunodeficiency virus (HIV), cancer, people on immunosuppressants, or people with chronic medical conditions like diabetes mellitus, epilepsy, stroke, congestive heart failure, coronary artery disease, chronic obstructive pulmonary disease, asthma, chronic liver disease, chronic kidney disease, and other medical conditions [6,10,12-13].

Introduction to myocarditis

Myocarditis is the inflammation of cardiac muscle and can be caused by infectious organisms, including viruses, bacteria, protozoans, toxins, autoimmunity, and hypersensitivity reactions. Viruses are the most common cause of myocarditis and account for about 50%-70% of all cases [14-16]. Influenza-myocarditis is the inflammation of the myocardium after influenza virus infection. Coxsackie viruses, adenoviruses, human herpes virus-6 type B, and parvovirus B19 are other causes of viral myocarditis [16]. Cardiovascular complications are the second most common cause of death due to influenza [17].

Influenza myocarditis clinical presentation and pathogenesis

Influenza myocarditis can have a varied clinical presentation with fever, myalgia, palpitations, shortness of breath, and chest pain to hemodynamic instability and collapse [18-20]. The 2017 National Healthcare Safety Network (NHSN) surveillance definition for myocarditis offers fair sensitivity and can be used even in resource-limited settings. Clinical features-based criteria for diagnosing myocarditis from NHSN requires (1) influenza identified from myocardial tissue or (2) ≥2 clinical features, including fever (>100.4°F), chest pain, paradoxical pulse, or increased heart size, with no other recognized cause plus ≥1 additional parameter, including an electrocardiogram (EKG) consistent with myocarditis, histological evidence of myocarditis, a four-fold rise in paired sera from the immunoglobulin G (IgG) antibody titer, or pericardial effusion [21]. Direct myocardial injury by the influenza virus and host cell immunity like increased expression of trypsin, matrix metalloproteinases, and cytokines like tumor necrosis factor (TNF) are thought to be the cause of influenza myocarditis and its complications but still the pathogenesis is largely unknown [18,22].

## Review

Methods

We searched Medline and Embase using relevant medical subject headings term ("Influenza virus" OR " flu virus" OR "Influenza" OR " flu" or "Flu vaccine" OR " influenza vaccine" OR "Influenza vaccination" OR "flu vaccine") and (" Myocarditis" OR " Myopericarditis" OR " Congestive heart failure" OR " cardiac disease" OR " congestive heart failure" OR "Cardiomyopathy" OR " arrhythmias" OR " heart failure" OR " sudden cardiac death" OR " cardiovascular mortality") for literature published till November 2020. The titles and abstracts of all results were reviewed, and studies were selected for full-text analysis according to the eligibility criteria.

Eligibility criteria

1. Studies focusing on myocarditis due to influenza virus were included for full-text analysis. We included adults as well as pediatric studies in our literature review due to the lack of enough studies. Studies focusing on cardiovascular complications due to influenza myocarditis like heart failure, arrhythmias were also included.

2. Clearly diagnosed influenza virus infection, e.g., culture- or polymerase chain reaction (PCR), serology, any other methods that confirmed influenza regardless of influenza viral types/subtypes with or without clinical features of influenza infection.

3. Exclusion criteria: Studies on languages other than English. We also excluded case reports in our study.

Outcomes of interest

The primary outcome of interest was influenza myocarditis. 

Quality assessment and data extraction

Two authors (Baral Nischit and Adhikari Prakash) independently performed the study selection, data extraction, and quality assessment.

Results and discussion

General Findings

We found that influenza A is the most common influenza virus-type associated with the highest cardiovascular morbidity and mortality among other influenza viral types [23]. Including all outbreaks, epidemics, and pandemics, the H1N1 strains of influenza A are the most common encounter followed by H3N2, H5N1, H7N9, and H2N2. Influenza B accounts for about 30%-40% of all influenza-related admissions and deaths, especially in the under 18 years of age group predominantly in the Asia-Pacific area [24]. Influenza C virus infection is less common than influenza A and B. It has a preference for young children aged below six years, and a total of 1%-2% of all influenza infections can be attributed to it. All of them have a similar clinical presentation but influenza C significantly shows more upper respiratory symptoms as compared to the other two [7].

**Figure 1:**
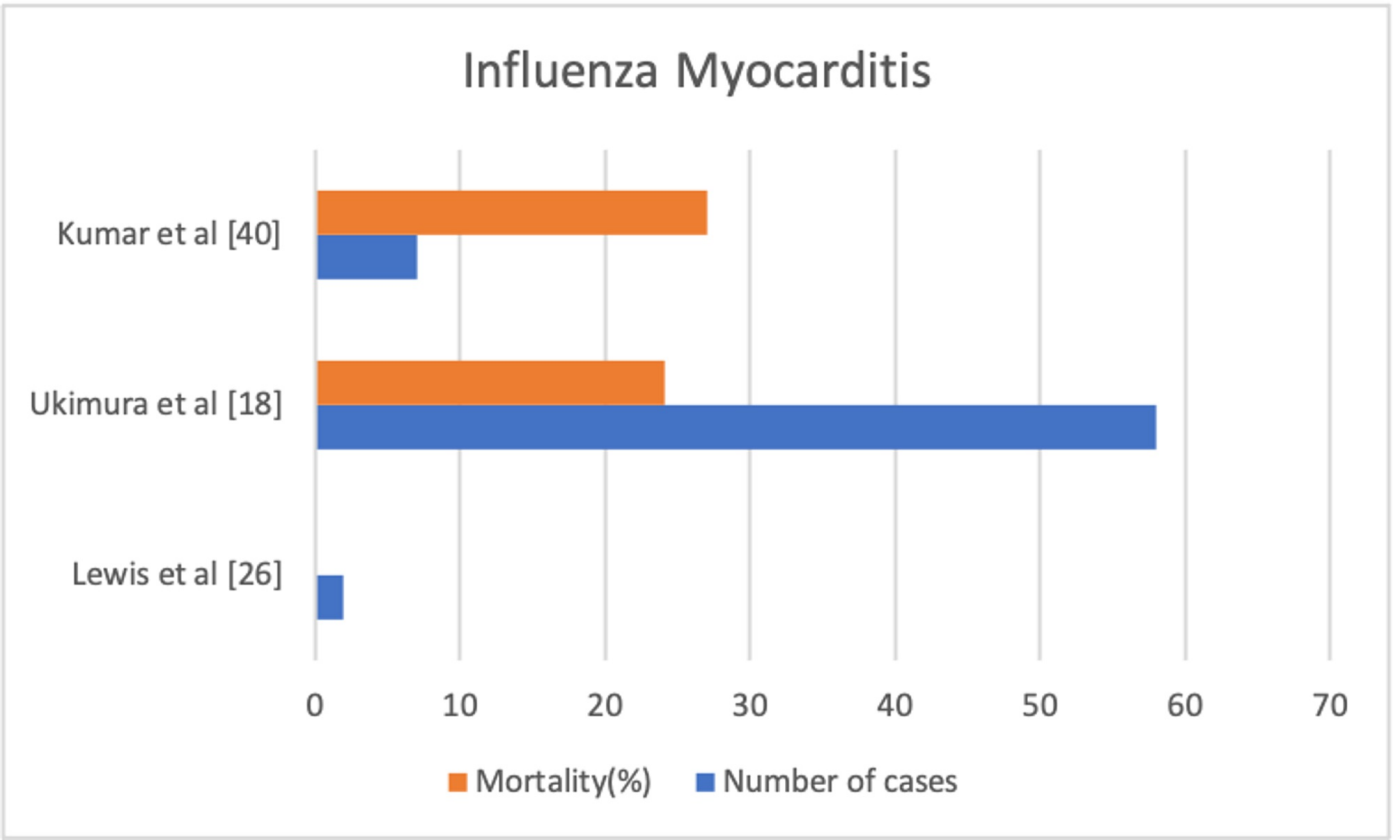
Studies on influenza myocarditis (mortality and number of cases)

EKG often shows sinus tachycardia, low amplitude QRS complexes, AV or bundle branch block, ST-segment changes, or even Q waves, many of which are non-specific findings. Most of the ECG changes are transient and reversible with the course of time [25-26]. Also, we found out that many of the ECG findings are contrasting between studies. A prospective study by Ison et al. and a retrospective study by Ukimura et al. showed ECG changes in the influenza patients but another prospective study by Kaji et al. in Japan showed normal ECG findings in these patients, which proves that ECG findings are inconsistent [19,27-28]. Elevated creatine kinase isoenzyme MB (CK-MB) fractions and cardiac troponins are also non-specific findings [18-20]. Echocardiography findings can vary from normal to focal or global hypokinesis, pericardial effusions, septal thickness, left, right, or combined ventricular dysfunction with low ejection fraction [29-30]. Endomyocardial biopsy (EMB) has variable sensitivity due to the chances of missing the involved site and the risk-benefit ratio of performing EMB being high [31]. Thus, we can come to the conclusion that clinic correlation is very important in influenza patients to diagnose influenza myocarditis [29-30]. Figure 1 graphically represents the findings of some studies on influenza myocarditis (mortality and number of cases).

Rapid diagnostic tests (RDT)/rapid antigen tests are often used earliest in clinical settings [32]. They are very specific but due to antigenic changes, their sensitivity can be a limiting factor. A meta-analysis done by Chu et al. estimated the sensitivity of RDT to be 0.51 (95% CI: 0.41-0.60) and specificity to be 0.98 (95% CI: 0.94-0.99) [33]. A study done by Greaves et al. reported that the prevalence of MC after an acute influenza infection is very low, whereas skeletal muscle injury is relatively common [34]. Though this may contrast with a study done by Lewes et al. where myocarditis and myalgia occurred in two out of seven patients with mild influenza infection, the very small sample size of Lewes et al. study warrants more studies to confirm the results and is misleading [26]. A study by Han et al. showed that out of 40 patients infected with lab-confirmed avian-influenza A H7N9, half of the patients presented with cardiovascular complications like hypotension (47.5%) and heart failure (40.0%) but the infection by H7N9 is common in birds and less common in humans [35].

Fulminant Myocarditis (FMC)

When myocarditis is associated with acute hemodynamic compromise and extensive myocardial inflammation, it is known as FMC. Aggressive management should be started immediately with ventilatory and circulatory support and guidelines-directed medical therapy for complications like heart failure and arrhythmias. Despite the fulminant presentation, high chances of mortality, and requirement of aggressive management, it has a high likelihood of complete recovery of ventricular function if the patient survives [18,29,36-39]. A study done by Ukimura et al. showed that during the H1N1 pandemic of 2009, among 58 total cases of influenza myocarditis, 36 patients had fulminant myocarditis and 14 (24.14%) patients with influenza myocarditis died [18]. A study by Kumar et al. reports seven total cases of influenza myocarditis during the 2009 H1N1 pandemic, with 27% mortality [40].

## Conclusions

Influenza myocarditis is a rare condition associated with influenza virus infection, and the complications of influenza myocarditis are even rarer. Fulminant myocarditis can be a fatal complication with high mortality so there should be increased awareness about influenza myocarditis. High clinical suspicion, especially in times of pandemic and the flu season, followed by early diagnosis and treatment can save lives.
